# Supplementary material for: An Innovative Inhibitor with a New Chemical Moiety Aimed at Biliverdin IXβ Reductase for Thrombocytopenia and Resilient against Cellular Degradation
Source: Pharmaceutics. 2024 Aug 30;16(9):1148. doi: 10.3390/pharmaceutics16091148 (PMC11435328; doi:10.3390/pharmaceutics16091148)
Supplement: Supplementary file 1 [file pharmaceutics-16-01148-s001.zip › pharmaceutics-3155149-supplementary.pdf]

pharmaceutics

MDPI

Supplementary Materials: An Innovative Inhibitor with a New Chemical Moiety Aimed at Biliverdin IXβ Reductase for Thrombocytopenia and Resilient against Cellular Degradation

Hoe-Myung Jung, Jung-Hye Ha, Mark Vincent C. dela Cerna, Joseph A. Burlison, Joonhyeok Choi Bo-Ram Kim, Jeong Kyu Bang, Kyoung-Seok Ryu and Donghan Lee

Stilbene Derivatives

Stilbene and its related motifs hold enormous potential applications due to their diverse spectrum of biological applications, such as anticancer, antiproliferative, antiangiogenesis, antimicrobial, antileukemic, and anti-HIV and, similarly, for industrial purposes, such as electrochemical, dyes, dye laser, coloring textiles, organic LED, fluorescent and optical brightener properties, etc.

Due to the nonavailability of naturally occurring stilbenes in adequate quantities, the development of new synthetic methodologies is required for their preparation on a large scale, like Wittig or Horner–Wadsworth–Emmons (HWE) olefination, Perkin aldol condensation, coupling reactions based on Suzuki–Miyaura, Sonogashira, Stille, Mizoroki–Heck, Negishi, Grubbs, McMurry, Knoevenagel–Doebner, Ramberg–Bucklund reactions, etc. Despite this, many synthetic strategies for the synthesis of stilbene scaffolds and related structures are available in the literature. Among them, we used a BBr3 (Lewis acid), which has been generally used for the demethylation of methyl aryl ethers.

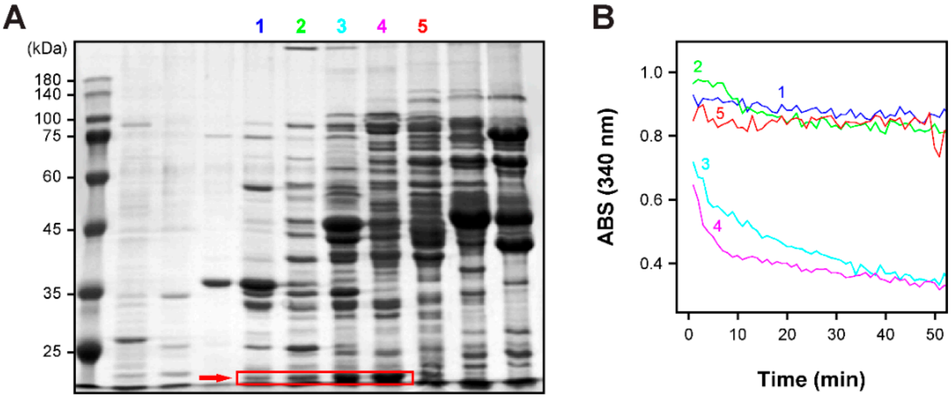

Figure S1. Partial purification and enzyme kinetics of AzoR enzyme: (**A**) SDS-PAGE analysis displaying the protein profiles after each purification step via anion-exchange chromatography under a gradient of NaCl. The red arrow and red box indicate the band presumed to be AzoR. (**B**) Enzyme activity assay of AzoR, as indicated by the decrease in absorbance at 340 nm over time, measuring the reduction of menadione by NADH in the eluted fractions. Each curve represents the activity corresponding to different fractions collected during the purification process.

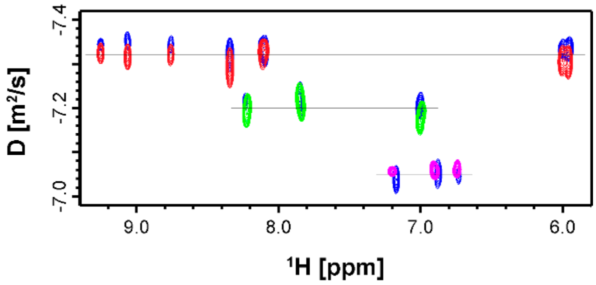

Figure S2. The DOSY spectra of olsalazine (green), NAD$^+$ (red), 5-ASA (magenta), and olsalazine treated with a cell extract (Figure S1) containing AzoR and NADH (blue).

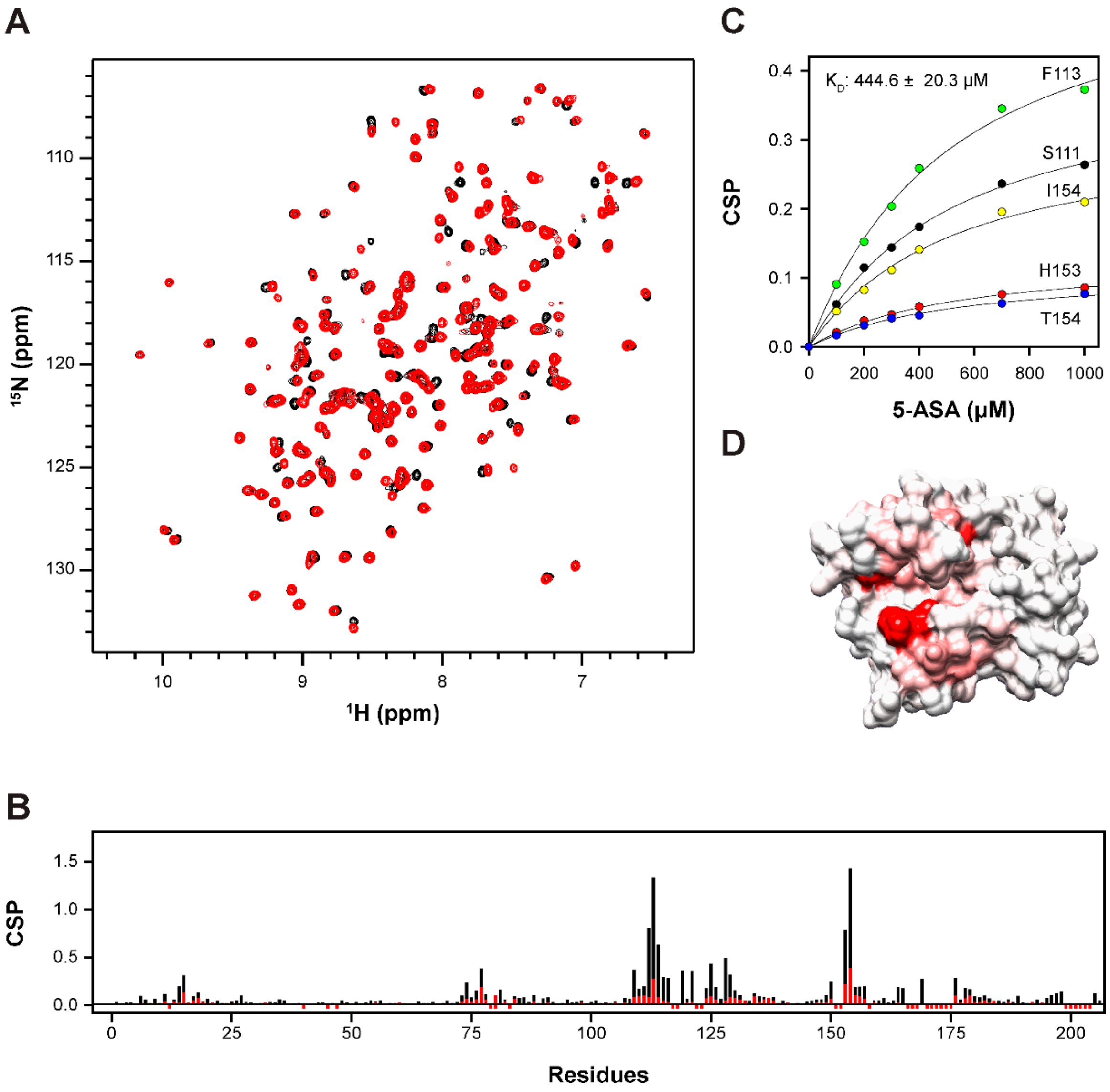

Figure S3. 5-ASA binding of half-holo BLVRB: (**A**) The ^{1}H-^{15}N HSQC spectra of 0.1 mM half-holo BLVRB in the absence (black) and presence (red) of 1 mM 5-ASA. (**B**) Chemical shift differences between half-holo BLVRB and BLVRB in complex with OSK (black) and 5-ASA (red). (**C**) NMR titration data for binding to half-holo BLVRB. (**D**) Surface representation of half-holo BLVRB with 5-ASA, generated using the crystal symmetry of the previously reported PDB structure (7ERA).

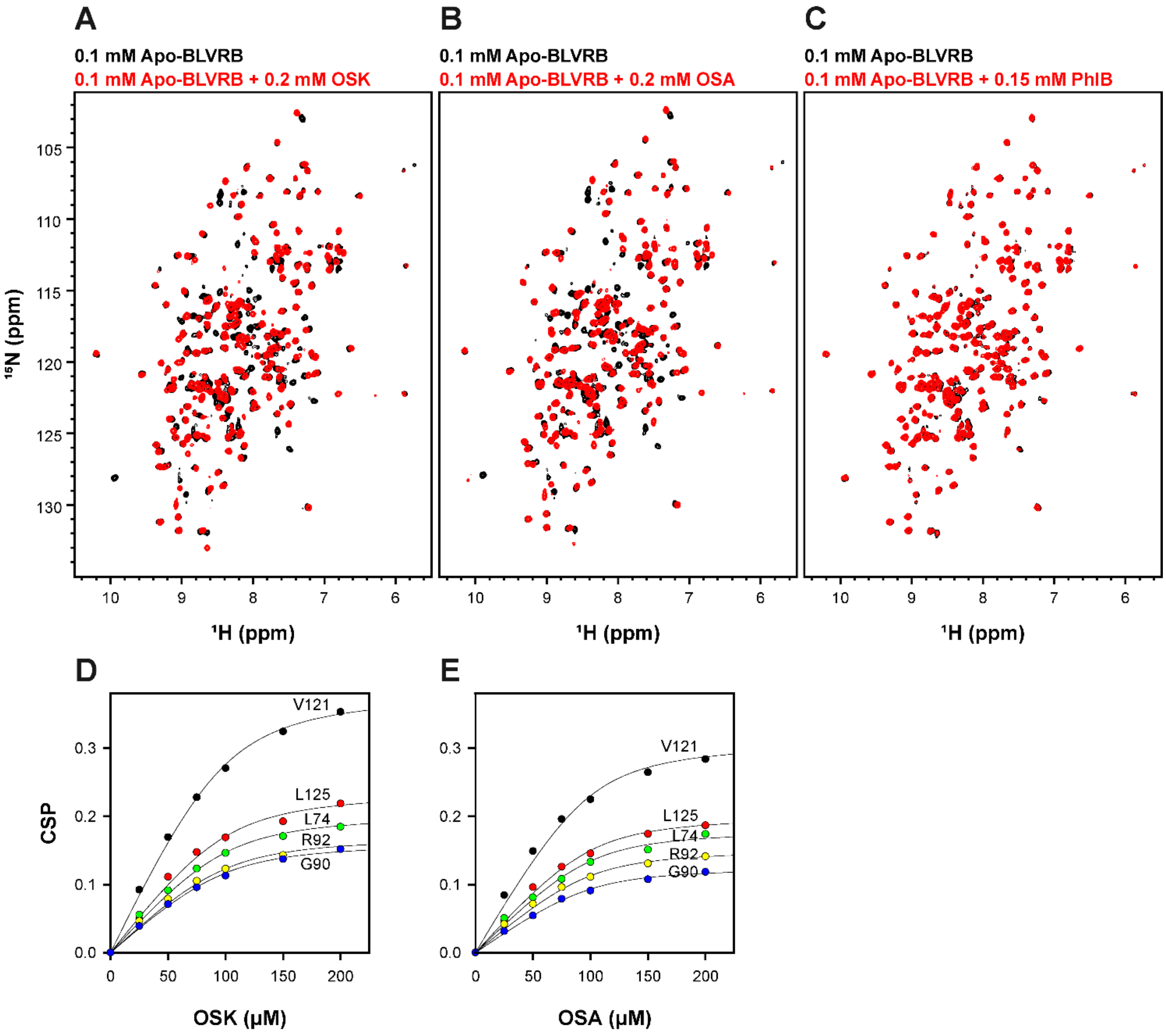

Figure S4. HSQC titration olsalazine and OSK bind to apo-BLVRB, but phloxine-B does not: The ^{1}H-^{15}N HSQC spectra of apo-BLVRB in the absence (black) and presence (red) of inhibitors (**A**) OSK, (**B**) OSA, and (**C**) PhlB are overlaid. NMR titration data for (**D**) OSK and (**E**) OSA binding to apo-BLVRB.

Table S1. Statistics of the X-ray data collection and structure refinement for the BLVRB:OCD complex.

**Data collection**	
**Synchrotron, beamline**	PAL, BL-5C
**Wavelength (Å)**	0.97957
**Resolution range (Å)**	33.9~1.7 (1.76~1.70)
**Space group & unit cell**	$P2_12_12$
**- *a*, *b*, *c* (Å)**	82.14, 117.30, 41.56
**- α, β, γ (°)**	90.0, 90.0, 90.0
**No. of total reflections**	417820 (27425)
**No. of unique reflections**	44771 (4381)
**Multiplicity**	9.3 (6.3)
**Completeness (%)**	99.86 (99.00)
**Mean I/σ (I)**	23.03 (3.82)
**R_{merge}**	0.0573 (0.254)
**Wilson *B* factor (Å^2)**	17.89
**Structure refinement**	
**No. of atoms**	3629
**- Protein/ligand/water**	3116/163/350
**No. of protein residues**	412
**R_{work}**	0.1612 (0.1810)
**R_{free}**	0.1930 (0.2311)
**RMSD bonds (Å)**	0.007
**RMSD angles (°)**	0.930
**Ramachandran favored (%)**	97.30
**Ramachandran outliers (%)**	0.74
**Clashscore**	2.61
**Average B factors (Å^2)**	
**- Overall**	24.71
**- Macromolecules**	23.51
**- Ligands**	27.48
**- Solvent**	34.09
**PDB deposit code**	8K4K
